# Priming with Small Molecule-Based Biostimulants to Improve Abiotic Stress Tolerance in *Arabidopsis thaliana*

**DOI:** 10.3390/plants11101287

**Published:** 2022-05-11

**Authors:** Alba E. Hernándiz, Carlos Eduardo Aucique-Perez, Sanja Ćavar Zeljković, Nikola Štefelová, Sara Salcedo Sarmiento, Lukáš Spíchal, Nuria De Diego

**Affiliations:** 1 Faculty of Sciences, Palacký University, Šlechtitelu 27, 78371 Olomouc, Czech Republic; 2 Centre of Region Haná for Biotechnological and Agricultural Research, Czech Advanced Technology and Research Institute, Palacký University, Šlechtitelu 27, 78371 Olomouc, Czech Republic; carloseduardo.auciqueperez@upol.cz (C.E.A.-P.); sanja.cavar@upol.cz (S.Ć.Z.); nikola.stefelova@seznam.cz (N.Š.); sara.salcedosarmiento@upol.cz (S.S.S.); lukas.spichal@upol.cz (L.S.); 3 Centre of the Region Haná for Biotechnological and Agricultural Research, Department of Genetic Resources for Vegetables, Medicinal and Special Plants, Crop Research Institute, Šlechtitelu 29, 78371 Olomouc, Czech Republic

**Keywords:** abiotic stress, growth, plant phenotyping, biostimulant

## Abstract

Biostimulants became a hotspot in the fight to alleviate the consequences of abiotic stresses in crops. Due to their complex nature, it is challenging to obtain stable and reproducible final products and more challenging to define their mechanism of action. As an alternative, small molecule-based biostimulants, such as polyamines have promoted plant growth and improved stress tolerance. However, profound research about their mechanisms of action is still missing. To go further, we tested the effect of putrescine (Put) and its precursor ornithine (Orn) and degradation product 1,3-diaminopropane (DAP) at two different concentrations (0.1 and 1 mM) as a seed priming on in vitro Arabidopsis seedlings grown under optimal growth conditions, osmotic or salt stress. None of the primings affected the growth of the seedlings in optimal conditions but altered the metabolism of the plants. Under stress conditions, almost all primed plants grew better and improved their greenness. Only Orn-primed plants showed different plant responses. Interestingly, the metabolic analysis revealed the implication of the N- acetylornithine and Orn and polyamine conjugation as the leading player regulating growth and development under control and stress conditions. We corroborated polyamines as very powerful small molecule-based biostimulants to alleviate the adverse abiotic stress effects.

## 1. Introduction

Climate change globally affects the earth’s temperature and rainfall pattern, causing extreme meteorological phenomena, amplifying drought events, their frequency, duration, and intensity [1,2]. One of the impacts of extended drought conditions is the significant increase in salinity in the root-zone soil, most likely occurring in irrigated areas but not exclusively [3]. These two phenomena, drought and salinity, already affect 60 and 10.5 million km^2^, respectively [4,5]. The entailed reduction of arable land becomes another obstacle to meeting the food demand. For that, food production is under threat due to exposure of the crops to numerous abiotic and biotic stresses that negatively affect plant growth and productivity, leading to reduced yields and quality [6]. This situation jeopardizes agricultural production and food security and entails long-term socioeconomic impacts. Consequently, the arable land availability will decline, so it is expected that the land for tropical and temperate crops will be reduced by 2.3 and 10.9%, respectively, in the year 2100. In addition, the prediction suggests that this reduction is expected to escalate up to 14.9% and 18.3% by 2500 [7].

Moreover, international organizations have alerted that the high food demand for a world population with a growth expectation of 9.1 billion by 2050 should increase by 50 to 70% of the current production [8]. Therefore, the climatic crisis and, consequently, the loss in food security are the target center of the world discussion, both in science and economic scenarios. In this context, drought has caught the attention of many researchers since water is a major component of the fresh biomass of the plants and plays a vital role in various physiological processes such as plant growth, development, and metabolism [9]. To counteract the adverse effects of drought on food production, scientific initiatives and research aim to improve the plant stress response through classic breeding strategies associated with genetic engineering tools to accelerate the obtaining of tolerant plants [10,11,12]. Concurrently, better agricultural practices have been developed to optimize the production systems and the study of the potential use of biostimulants as products that might contribute to easing the enormous pressure that climatic factors are putting on agriculture. According to the Regulation (EU) 2019/1009 of the European Parliament, a biostimulant is “a product stimulating plant nutrition processes independently of the product’s nutrient content, with the sole aim of improving one or more of the following characteristics of the plant or the plant rhizosphere: nutrient use efficiency, tolerance to abiotic stress, quality traits, and availability of confined nutrients in soil or rhizosphere”. Under this definition, there are comprised a wide range of substances that are currently classified according to their source of origin [13], providing the following categories: humic and fulvic acids, seaweed and botanical extracts, protein hydrolysates, and N-containing compounds, chitosan and other biopolymers, inorganic compounds and beneficial fungi, and bacteria. Biostimulants are commonly a mixture of several substances since the circular economy has seen in this sector a niche to give a second life to waste and by-products [14,15]. Nevertheless, the use of mixtures presents some disadvantages, such as the possible lack of homogeneity of the batches and the difficulty in identifying the active substances [16]. Therefore, there are limitations to studying and accurately describing the mode of action of those substances to prove their effectiveness, but it is a challenge that can be overcome considering the new technologies that exist.

Another possibility is the use of small molecule-based biostimulants. They are natural compounds such as amino acids, carbohydrates, polyamines, plant growth regulators, or other nanoparticles, which can be easily found in plants and help them deal with stress [17,18]. They are also involved in plant–microorganism communication [19], giving the plant higher tolerance to abiotic stress or resistance to biotic stress [18,20]. Our previous works demonstrate that priming Arabidopsis seeds with small molecules such as polyamines, especially Put and Spd, improves plant growth under optimal conditions and increases salt stress tolerance. However, the mechanism of action that the seed priming with these compounds activates in the plant is still unclear. To go further in this issue, we decided to study a precursor [ornithine (Orn)], a product [putrescine (Put)], and a final product [1,3-diaminopropane (DAP)] of the polyamine (PA) metabolism.

Orn is an amino acid produced from glutamate (Glu), essential in nitrogen assimilation metabolism. Orn participates in the arginine (Arg) synthesis, the urea cycle, and as a precursor of many stress-related compounds, including PAs synthesis and the amino acids proline (Pro) and γ-aminobutyric acid (GABA), which, in turn, is linked to the tricarboxylic acids cycle (TCA) [21]. Only one publication reported the beneficial effect of the exogenous Orn application on beetroot plants subjected to drought stress [22]. Put is the first to be PA synthesized, and it has been extensively reported for its positive contributions to the plant response to several stresses, improving the photosynthetic capacity, contributing to the enhancement of amino-oxidases, maintaining the membrane stability, contributing to reactive oxygen species (ROS) scavenging mechanisms, or enhancing plants growth and germination [23,24,25]. The Put catabolism can end with the DAP formation. This diamine has been reported to repress ethylene biosynthesis [26]. In addition, its acetylated product diacetyl-DAP, originated by the action of the *N*-acetylaminotransferase 1 (NATA1, E.C 2.6.1.11.) enzyme, has been described as an antagonist of the abscisic acid (ABA)-regulated stomatal closing [27]. However, no studies exist about its effects as a priming agent to mitigate the adverse stress effects.

Both Orn and DAP are directly linked with metabolic pathways that control plant stress tolerance. We hypothesize that the exogenous application with DAP and Orn as small molecule-based biostimulants could be a simple and efficient approach for improving plant stress tolerance. To validate our hypothesis, deep characterization of DAP, Orn, and Put as seed priming agents was performed using *Arabidopsis thaliana* plants growing in vitro under optimal conditions or subjected to osmotic and salt stresses based on [28]. In addition, the combination of phenomic and metabolomics data will uncover the critical metabolic steps in which these metabolites affect and condition the Arabidopsis phenotype under the different growth conditions studied.

## 2. Results

### 2.1. The Priming with DAP, Orn, and Put Improved Plant Performance under Abiotic Stress but Not under Optimal Growth Conditions

**Figure 1:**
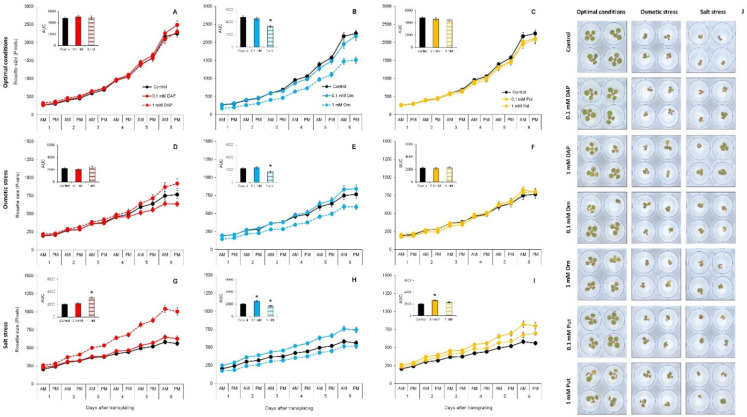
Rosette growth (green pixels, left panel) of *Arabidopsis thaliana* (Col-0 ecotype) primed with 1,3-diaminopropane (DAP), Ornithine (Orn), or Putrescine (Put) at two concentrations (0.1 or 1 mM) under optimal conditions (**A**–**C**), osmotic stress (**D**–**F**), and salt stress (**G**–**I**). RGB images (**J**) of the Arabidopsis seedlings 7 days after the transfer to the well-plates with different growth conditions. The corresponding area under the curve (AUC) for each growth curve was calculated and displayed in the bar chart. According to Kruskal–Wallis’ tests, asterisks show significant differences compared to the respective control treatment (*p* < 0.05). Bars correspond to standard error. *n* = 48. Well diameter = 10.4 mm.

As the first step for studying DAP, Orn, and Put as priming agents, we analyzed the Arabidopsis rosette growth under optimal conditions and osmotic and salt stress. Osmotic and salt stress reduced 70% and 78%, respectively, the rosette size of Arabidopsis compared to the seedlings grown under control conditions (Figure 1). The seed priming with DAP, Orn, and Put modified the growth compared to the seedling from non-priming seeds, but the effect depended on the interaction between the compound concentration and growth condition. For example, the priming with DAP and Put did not alter Arabidopsis rosette growth under optimal conditions or osmotic stress, but they improved it under salinity (Figure 1). The low Orn concentration also improved plant growth under salt stress. Contrarily, 1 mM Orn reduced the rosette growth under optimal and stress conditions (Figure 1B,E,H).

**Figure 2:**
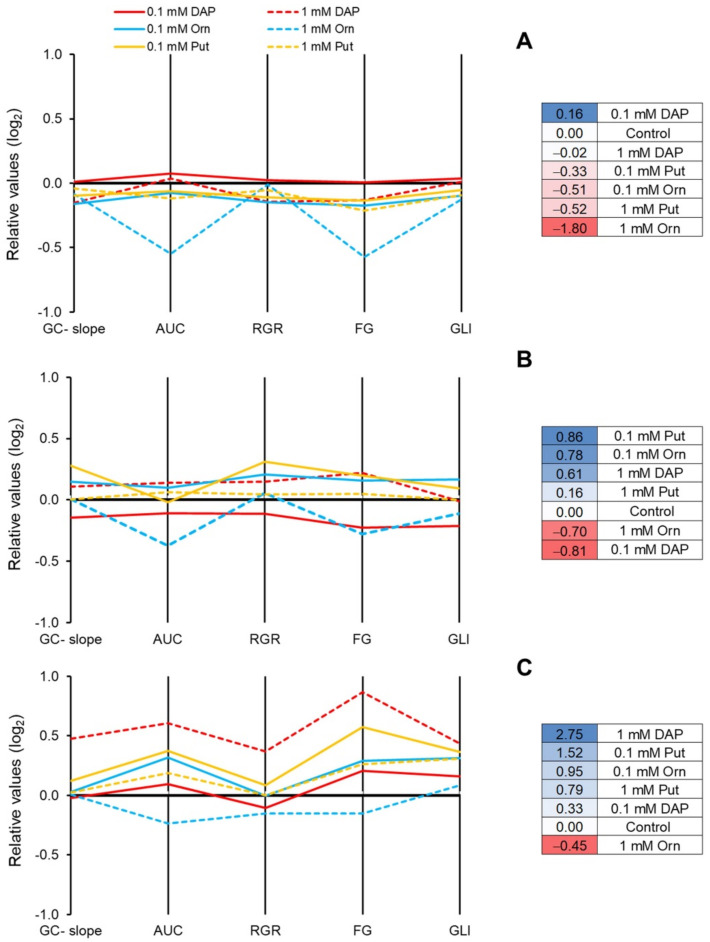
Parallel coordinates plot (left panel) representing all the phenotyping traits; the slope of the growth curve (GC-slope), the AUC of the growth curve, the relative growth ratio (RGR), the final size of the seedlings at the end of the experiment (FG), and the green leaf index (GLI) in Arabidopsis seedlings from primed seeds with DAP, Orn, or Put at two concentrations (0.1 or 1 mM) grown under optimal growth conditions (**A**), osmotic (**B**), and salt stress (**C**). Plant Biostimulant Characterization Index (PBCI) (right panel) is calculated as the sum of the values represented for each phenotyping trait in the parallel coordinates plot. The blue color indicates plant growth promotor under optimal conditions or stress alleviator under stress. The red color indicates a stress inductor.

The phenotyping traits extracted from the RGB images were represented in a parallel coordinate plot (Figure 2, left panel) [29]. The obtained value per trait is recalculated using log2 and represented in a parallel coordinate plot (Figure 2, left panel) [29]. Finally, the values for each trait are summed individually per treatment and growth condition to obtain the PBCI, which is included in an independent table in the right panel of Figure 2. The treatments presenting positive values will be considered plant growth promotors under optimal conditions or stress alleviators under stress conditions. Contrarily, the negative values will describe the stress inductors.

For the optimal growth conditions, none of the treatments enhanced the plant performance for the studied parameters but instead had a negative effect, ending with negative values of the PBC index for all the treatments (Figure 1A). The only plant growth promotor was 0.1 mM DAP, presenting a positive PBCI due to a better relative growth ratio (RGR) and the green leaf index (GLI) (Figure 2A and Supplementary Table S1).

Although no apparent effect was observed in the growth curves of the seedlings from primed seed compared to their controls under osmotic stress, a deeper analysis in which parameters such as RGR, final size, or the color index were extracted showed a positive effect on the plants (Figure 2B). The seed priming with 1 mM DAP and 0.1 mM Orn provided the highest values of PBCI and the best seedling performance. The Put application slightly improved the plant performance (Figure 2B). However, 1 mM Orn and 0.1 mM DAP showed negative PBCI values due to decreased traits such as the slope of the curve, RGR, final rosette size, or GLI (Figure 2B and Supplementary Table S1).

All treatments alleviated the salt stress adverse effects (Figure 2C). Only 1 mM Orn showed a negative PBCI, but it improved GLI compared to the non-primed plants. The best salt stress alleviator was 1 mM DAP, which greatly enhanced all phenotype parameter. Similar trends were observed in the rest of the treatments, with 0.1 mM DAP as the less effective stress alleviator (Figure 2C).

### 2.2. Seed Priming Induced Changes in the N-Related Metabolites, and the Changes Were Due to the Interaction between the Priming Agent, Its Concentration, and Growth Conditions

**Figure 3:**
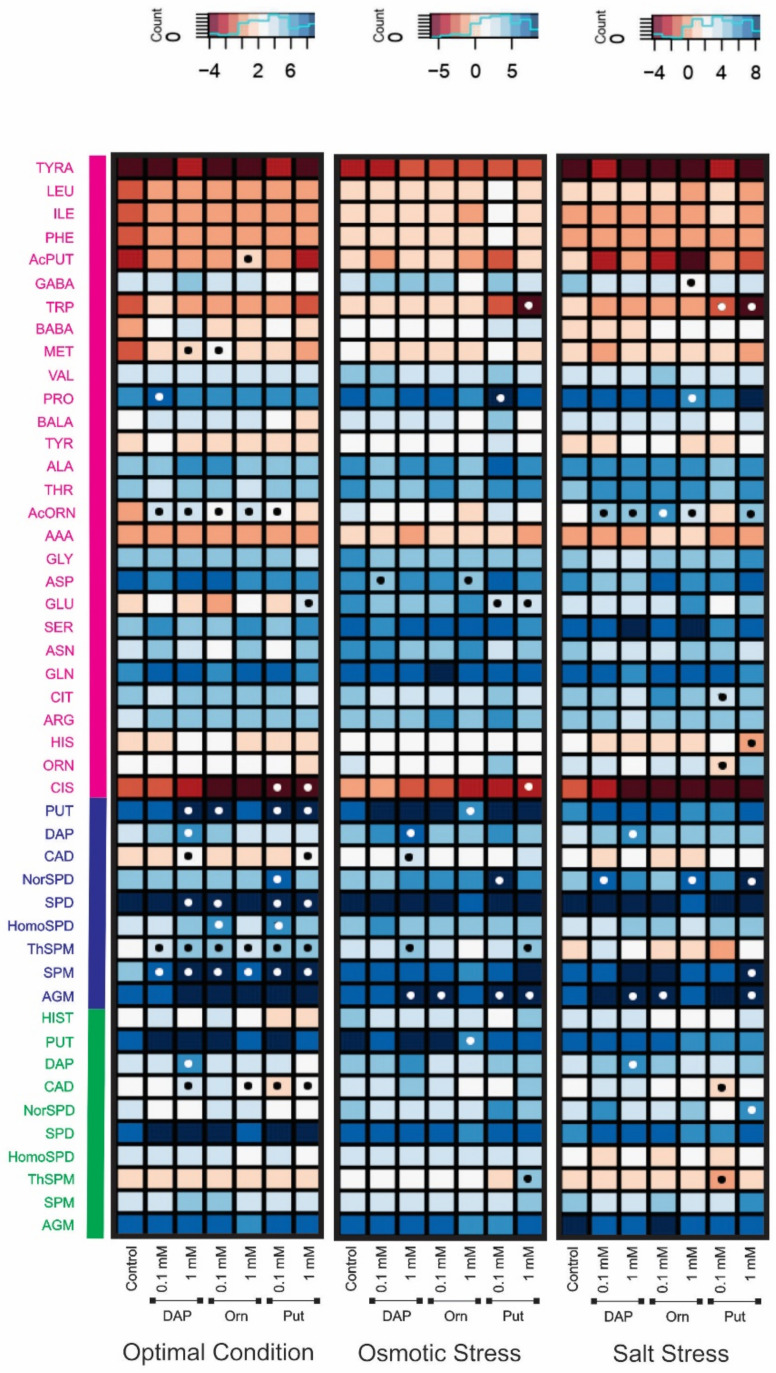
Metabolic profile of free amino acids (pink) [tyramine (TYRA), leucine (LEU), ileucine (ILE), phenylalanine (PHE), N-acetylputrescine (AcPUT), γ-aminobutyric acid (GABA), tryptophan (TRP), β-aminobutyric acid (BABA), methionine (MET), valine (VAL), proline (PRO), β-alanine (BALA), tyrosine (TYR), alanine (ALA), threonine (TRH), N-acetylornithine (AcORN), 2-aminoadipic acid (AAA), glycine (GLY), aspartic acid (ASP), glutamate (GLU), serine (SER), asparagine (ASN), glutamine (GLN), citruline (CIT), arginine (ARG), histidine (HIS), ornithine (ORN), cystine (CIS)], total polyamines (blue) [putrescine (PUT), 1,3-diaminopropane (DAP), cadaverine (CAD), norspermidine (NorSPD), spermidine (SPD), homospermidine (HomoSPD), thermospermine (ThSPM), spermine (SPM), agmatine (AGM)], and free polyamines (green) [histamine (HIST), PUT, DAP, CAD, NorSPD, SPD, HomoSPD, ThSPM, SPM, AGM] in *Arabidopsis thaliana* (Col-0 ecotype) seedlings from primed seeds with 1,3-diaminopropane (DAP), Ornithine (Orn), or Putrescine (Put) at two concentrations (0.1 or 1 mM) grown under optimal conditions, osmotic stress, and salt stress. Data were normalized through a natural logarithm. Each cell represents the values of four biological replicates (*n* = 4). Red and blue colors indicate a decrease and increase, respectively, for the level of each metabolite. Mean values containing black or white points show significant differences among treatments evaluated by Tukey’s test (*p* < 0.05).

We analyzed the metabolite changes on the rosettes collected after the last phenotyping measurement in the following step. Due to the involvement of Orn, Put, and DAP in the amino acid and PA metabolism, we performed a targeted metabolomic analysis for their quantification (Figure 3 and Supplementary Materials Table S2). The most abundant free amino acids were Pro, aspartic acid (Asp), and glutamine (Gln) in Arabidopsis plants from all the treatments and growth conditions (Figure 3 and Supplementary Table S2). Contrarily, cystine (Cis) was the amino acid that appeared in the lowest concentration (Figure 3). Although there were not many significant changes in the content of free amino acids, some of them changed by the effect of the treatment and the growth conditions (Figure 3). For example, all primed plants accumulated N-acetylornithine (AcOrn) under optimal and salt stress conditions, except for the 1 mM or 0.1 mM Put-treated plants under optimal or salt stress conditions, respectively. Met was significantly accumulated with 1 mM DAP and 0.1 mM Orn under optimal conditions. Only the primed seedlings with 1 mM DAP or 0.1 mM Put significantly accumulated Pro under optimal and osmotic stress conditions, respectively. However, 1 mM Orn reduced the Pro content. Glu was only significantly accumulated in 1 mM Put-primed plants and reduced in Put-treated ones under osmotic stress (Figure 3). The Put-treated seedlings also reduced the content of Cis under optimal conditions and in the high concentration under osmotic stress. Finally, the 0.1 mM DAP or 1 mM Orn reduced the levels of Asp under osmotic stress (Figure 3).

Regarding PAs, both free and total (sum of conjugated and free PAs) Put, Spd, and agmatine (Agm) were the most abundant in all plants, whereas tyramine (Tyra) appeared in low concentrations (Supplementary Table S3). Only 1 mM DAP, 0.1 mM Orn, and both Put concentrations induced significant increments for the total forms under optimal conditions (Figure 3, left panel). All primed plants presented higher total thermospermine (ThSpm) and spermine (Spm) levels than controls under optimal conditions. Total ThSpm was also accumulated in 1 mM DAP and Put-primed seedlings under osmotic stress. They also accumulated higher total Cadaverine (Cad) levels but reduced the free form under optimal conditions (Figure 3, left panel). Finally, the uncommon PA homospermidine (HomoSpd) was more abundant in the Arabidopsis seedlings from 1 mM Orn and Put-primed seeds than in the untreated plants under optimal conditions. However, the 0.1 mM Put-treated plants accumulated norspermidine (NorSpd) under optimal conditions, osmotic stress, and salt stress when plants were primed with 0.1 mM DAP and 1 mM Orn and Put (Figure 3). It is worth mentioning that the acetylated form AcPut significantly increased in 0.1 mM Orn treated plants under optimal conditions (Figure 3).

### 2.3. Seed Priming Induced Changes in the N-Related Metabolites, and the Changes Were Due to the Interaction between the Priming Agent, Its Concentration, and Growth Conditions

**Figure 4:**
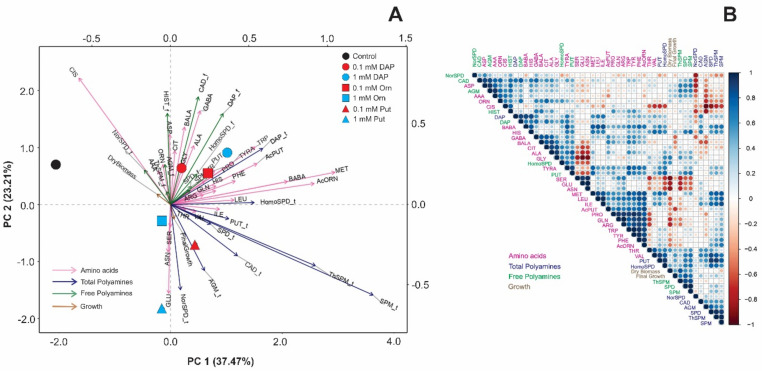
Principal components analysis (PCAs) (**A**) and correlation matrix (**B**) of the N-containing metabolites [free amino acids (pink arrows), total polyamines (blue arrows), free polyamines (green arrows)] and growth (dry biomass and final growth) in *Arabidopsis thaliana* (Col-0 ecotype) seedling from unprimed (control, black circle) or primed seeds with DAP (circles), Orn (square), or Put, (triangle) at two concentrations [0.1 mM (red symbols) or 1 mM (blue symbols)] grown under optimal conditions. Data were normalized using the natural logarithm. Each geometric figure (circle, square, and triangle) represents the mean values of four biological replicates (*n* = 4). Blue and red colors in the correlation matrix indicate positive and negative values, respectively.

**Figure 5:**
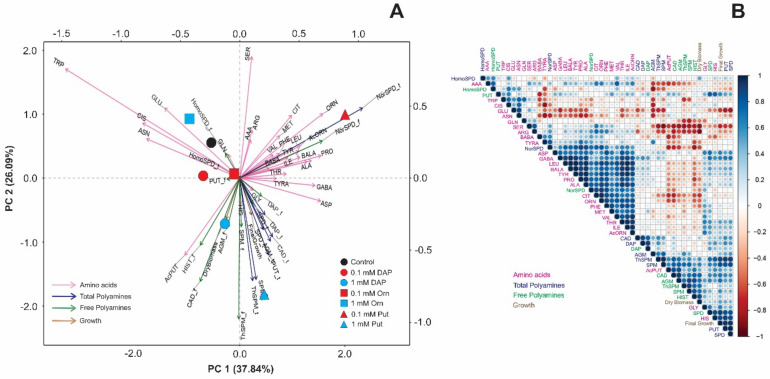
Principal components analysis (PCAs) (**A**) and correlation matrix (**B**) of the N-containing metabolites [free amino acids (pink arrows), total polyamines (blue arrows), free polyamines (green arrows)] and growth (dry biomass and final growth) in *Arabidopsis thaliana* (Col-0 ecotype) seedling from unprimed (control, black circle) or primed seeds with DAP (circles), Orn (square), or Put (triangle) at two concentrations [0.1 mM (red symbols) or 1 mM (blue symbols)] grown under osmotic stress. Data were normalized using the natural logarithm. Each geometric figure (circle, square, and triangle) represents the mean values of four biological replicates (*n* = 4). Blue and red colors in the correlation matrix indicate positive and negative values, respectively.

**Figure 6:**
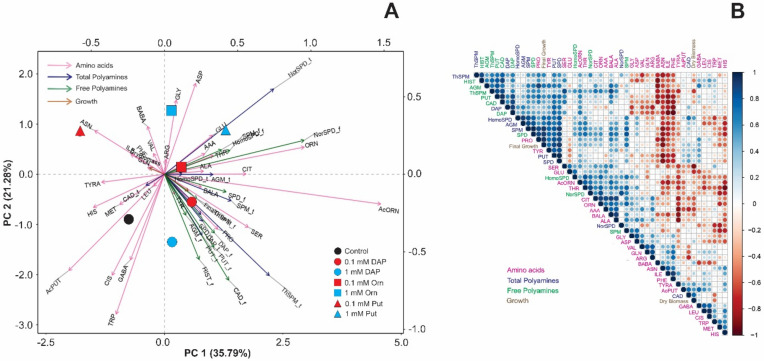
Principal components analysis (PCAs) (**A**) and correlation matrix (**B**) of the N-containing metabolites [free amino acids (pink arrows), total polyamines (blue arrows), free polyamines (green arrows)] and growth (dry biomass and final growth) in *Arabidopsis thaliana* (Col-0 ecotype) seedling from unprimed (control, black circle) or primed seeds with DAP (circles), Orn (square), or Put, (triangle) at two concentrations [0.1 mM (red symbols) or 1 mM (blue symbols)] grown under salt stress. Data were normalized using the natural logarithm. Each geometric figure (circle, square, and triangle) represents the mean values of four biological replicates (*n* = 4). Blue and red colors in the correlation matrix indicate positive and negative values, respectively.

To better visualize and integrate the metabolomic with the morphologic results, we performed three principal component analyses (PCA) and correlation matrices individually for each growth condition (Figure 4, Figure 5 and Figure 6). The two first PCs explained 60.68% (PC 1 = 37.47%; PC 2 = 23.21%, for optimal conditions), 63.93% (PC 1 = 37.84%; PC 2 = 26.09%, osmotic stress), and 57.07% (PC 1 = 35.79%; PC 2 = 21.28%, salt stress) of the total model variation. Under optimal conditions, the control treatment was related to Cis, NorSpd, and dry biomass, whereas Put-primed plants positively correlated with final growth, Glu, and total PAs, especially NorSpd and Agm (Figure 4A). Control plants were also located opposite the acetylated compounds N-acetylputrescine (AcPut) and AcOrn. Contrarily, DAP-treated plants correlated with almost all free amino acids and PAs, including Pro (Figure 4A). The accumulation of Pro also presented a negative correlation with the dry biomass. However, this morphological parameter and the final growth were positively correlated with the free ThSpm, Spm, and spermidine (Spd). Finally, the accumulation of free amino acids such as Glu, serine (Ser), asparagine (Asn), Arg, and Gln was negatively correlated to the final growth (Figure 4B). Altogether, the priming agents that induced the accumulation of free amino acids, especially Pro, and those related to Glu metabolism reduced free PAs levels like Spd and limited plant growth under optimal conditions.

Under osmotic stress, the metabolic profile in unprimed or primed Arabidopsis seedlings showed substantial alterations compared to optimal conditions (Figure 5). Whereas the seedlings from 0.1 mM Put-primed seeds accumulated the highest number of free amino acids, the free and total NorSpd, 1 mM Put, and DAP-treated plants showed higher total and free PAs and bigger final growth and dry biomass (Figure 5A). The rest of the treatments were located close to the controls, positively correlated to Cis, tryptophan (Trp), Glu, and the total and free HomoSpd. Similar results were obtained in the correlation matrix, whereas a strong correlation was observed between the final growth and the total Put, Spd, ThSpm and Spm, free Put, and the free amino acids GABA and Asp but less Glu and Ser. Dry biomass positively correlated with free PAs but negatively with Cit, Orn, Arg, Ser, and NorSpd (Figure 5B).

Different spacial distribution was observed in the PCA performed for the Arabidopsis seedlings grown under salt stress (Figure 6). For example, the controls correlated with GABA, methionine (Met), Cis, and AcPut. Their response was opposite to 1 mM Put-primed seedlings that accumulated higher levels Glu and Orn, free and total HomoSpd and NorSpm, the free Spm. Meanwhile, the DAP treatment was related to Pro, almost all the PA forms (free and total), including Spd and Cad, and the plant biomass (Figure 6). The correlation between high levels of Pro, Cad, and Spd with the final biomass was then corroborated by the correlation matrix with a highly significant linear correlation (Figure 6B). Contrarily, the accumulation of β-aminobutyric acid (BABA), Asn, isoleucine (Ile), phenylalanine (Phe), Met, and histidine (His) was opposite to the final growth (Figure 6B). Altogether, it is clear that the effectiveness of the seed priming with Orn, Put, and DAP for improving plant growth and stress tolerance depends on the type of growth conditions.

## 3. Discussion

Seed priming has earned recognition as an innovative and affordable technology to counteract the harmful effects of abiotic stress since it enhances the plant defense responses [30]. The use of natural compounds or biostimulants as priming agents has shown promising results in improving plant performance under suboptimal conditions [29,31]. The use of these substances is more sustainable and environmentally friendly compared with the use of other materials. In addition, some of them regulate plant growth, development, and health by controlling plant–microorganism interaction [18,19]. In this context, studying the mechanism and mode of action of pure substances or complex formulations with biostimulant potential is essential to describe better the plant defense process, standardize formulations, and assist the development of new products by the agrochemical industry [32]. Moreover, priming causes changes in the growth pattern and alterations in the plant metabolism and, hence, in the plant stress tolerance and resistance [33,34]. Plant phenotyping platforms are valuable tools for characterizing plant biostimulants or other priming agents, so they can quickly scan a multitude of variables and/or parameters, allowing a high-throughput pipeline [35,36]. Previous studies performed in the XYZ system (Olophen phenotyping platform) have already been proven as an accurate method to evaluate the efficiency of the seed priming for our in vitro assays based on *A. thaliana* rosette growth [29,31]. Using our previously described approach, we evaluated the use of small molecules-based biostimulants by applying DAP, Orn, or Put as priming agents to improve plant growth and tolerance. As a result, no concrete profile was observed in the primed plants because the response changed due to the interaction effect between the compound, the concentration used, and the growth conditions.

Under optimal conditions, the seed priming did not affect the plants (Figure 3). We speculated that the seed treatment induced a hardening process to prepare the plant for future stress situations or activate a plant eustress response [37] to become a more efficient organism (less energy expended and more growth). To solve these two possible hypotheses, we analyzed the content of free amino acids due to their involvement in plant stress response [38,39]. However, only the AcOrn was significantly increased in all primed plants, except those with 1 mM Put, compared to the controls. There is not much information about the biological relevance of this non-protein amino acid. Only a few studies have linked this metabolite as nitrogen storage or a precursor of the Orn biosynthesis [40,41]. Recently, it has also been related to the plant stress response. For example, [42] observed the AcOrn accumulation in a heat stress-tolerant and sensitive wheat lines under heat stress alone or combined with drought. It could be that plants accumulate AcOrn under stress as part of their plant defense, so it has been reported to promote the synthesis of methyl jasmonate in Arabidopsis [40]. Similar results were observed in our work because the unprimed Arabidopsis seedlings showed higher levels of this metabolite when grown under stress conditions (Figure 3, Supplementary Table S2). In addition, the seed priming enhanced this accumulation in all treated plants under salt stress, except for those with 0.1 mM Put, but not under osmotic stress. One possible explanation is that under salt stress, the plants suffer double stress induced by the osmotic pressure of the solute concentration and the toxic effect of the ion accumulation [31]. This was mainly visible in the plant growth, with the most miniature plants obtained under salt stress conditions. It is worth mentioning that the 1 mM Put-primed plants did not accumulate significant levels of AcOrn under optimal conditions but increased the Glu content (Figure 3 and Supplementary Table S2). Moreover, the Arabidopsis seedlings primed with 0.1 mM Put did not show significant changes of AcOrn compared to the unprimed ones under salt stress and reduced their levels of Orn and Cit. Considering that AcOrn is part of the Glu metabolism to produce Orn [43] and that both Cit and Orn are precursors of the PA biosynthesis, we could conclude that their changes are relevant players regulating plant growth and stress response.

Regarding Pro, it is the most stress-related amino acid in plants and is highly accumulated under stress conditions [24]. This metabolite is considered an osmoprotectant and regulator of the plants’ cellular homeostasis, redox balance, and energy status. In our study, the most significant accumulation of Pro occurred mainly in the best performing variants; the plants primed with 0.1 mM DAP under optimal conditions and 0.1 mM Put under osmotic stress. Contrarily, 1 mM Orn reduced it and GABA content. As [24] described, the metabolism of GABA, Pro, and PAs is closely connected to regulating plant growth and development. Their interconversion activates many biological processes in the plants, with the antioxidative response as the main one under stress conditions. Altogether, we could conclude that the seed priming with the polyamines Put and DAP activate the accumulation of stress-related amino acids under optimal conditions, most likely to activate the antioxidative response of the plant to deal with the following stressful situation. In addition, this accumulation permits the plant to perform better under abiotic stress, ending with better plant growth. However, the most exciting phenotype was observed in 1mM Orn-treated plants that showed a negative PBCI due to the inhibition of growth under salt stress. However, they improved the greenness GLI, the levels of AcOrn, and total NorSpd but reduced GABA and Pro. Further studies will be needed to explain the contrasting response of these plants.

The seed priming also affected the PA content of PAs (Figure 3). The most significant changes were observed for the total content of ThSpm and Spm, followed by the total Put content under optimal conditions. However, the primed plants grown under stress conditions mainly accumulated total Agm. An increase in the total content of specific PAs but not in their free forms pointed to a relevant increase in conjugation as a primary consequence of the seed priming. Although very little is known about the biological relevance of the conjugation PAs, [44] recently reviewed the current state of the art. They described that PAs could be conjugated with phenolic compounds forming a class of secondary metabolites called phenolamides or hydroxycinnamic acid amides (HCAAs). These metabolites play a critical role in plant growth and developmental processes, including cell division, cytomorphogenesis, flowering, cell wall cross-linking, tuberization, and stress responses [44,45,46]. As an example of their implication in the plant stress response, it has been shown that p-coumaroylagmatine, the major HCAA accumulated in Arabidopsis, inhibits *Phytopthora infestans* spore germination in vitro [47]. However, the increase of p-coumaroylagmatine and feruloylagmatine have also been identified in Arabidopsis leaves after infection with the pathogen *Alternaria brassicicola* [48]. One possible explanation of these controverted results is that the plant could perceive the presence of the pathogen and activate the jasmonic acid (JA)/ethylene (ET) signaling pathways that, at the same time, activate the transcription factor ORA59, which can bind the promoter of an Arabidopsis agmatine coumaroyl transferase (AtACT) and enabled its expression and HCAAs biosynthesis [49]. Thus, enhancing the pool of conjugated PAs in Arabidopsis seedlings from primed seeds could be clear evidence of stress response and, hence, plant hardening for better performance under future stress conditions.

To further this idea, we investigated the possible role of the conjugated forms with Spm or ThSpm. However, no HCAAs connected to Spm or ThSpm have been detected in Arabidopsis. This means that the high increase of their conjugated forms could have a different role. It has been reported that PAs can also bind particular proteins as post-translational regulators, and the induced modifications may have a specific functional role under concrete growth conditions [50]. Recently, [51] reviewed the interaction of the main PAs, Spm, Spm, and ThSpm, with the translation machinery of the plants.

## 4. Conclusions

**Figure 7:**
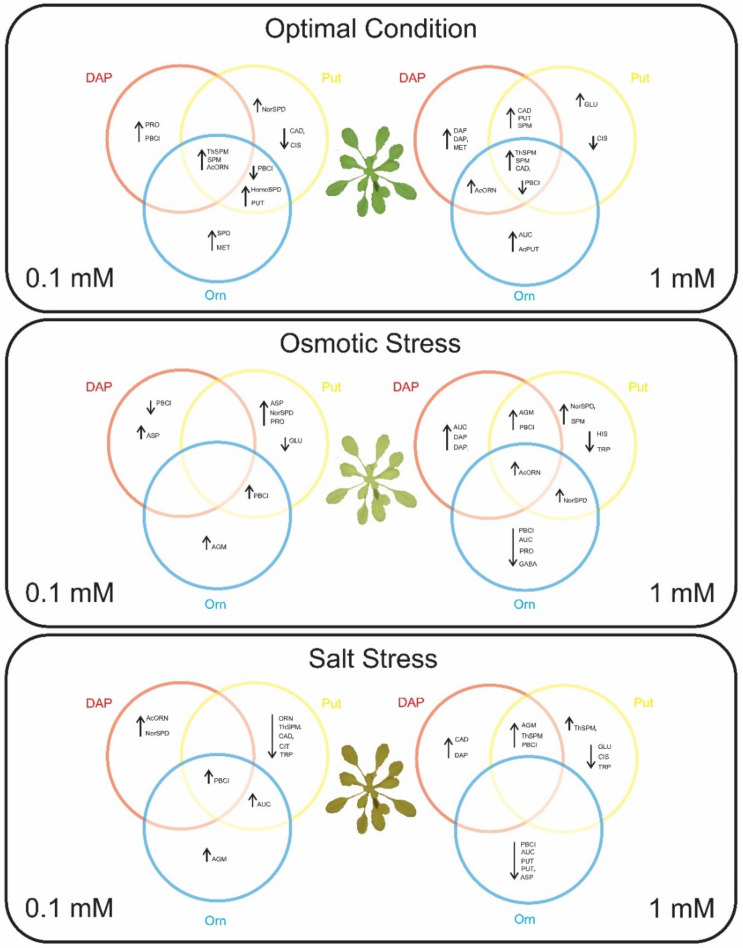
Summary of the reported effects of the seed primings with DAP, Orn, and Put at 0.1 and 1 mM on the *Arabidopsis thaliana* seedlings exposed to optimal growing conditions, osmotic, and salt stress. Red circles are for DAP, blue circles are for Orn, and yellow circles are for Put; on the left side of the plant is represented the lower concentration priming, while on the right is represented the higher priming concentration. Inside the circles, significant changes regarding growth (AUC), the potential biostimulant effect (PBCI), free and total polyamines, and free amino acids are represented. An upside arrow indicates significant increment, while a downside arrow means significant reduction compared to the unprimed treatment within the same growing condition. Significant factors situated in the intersection of two or all primings mean that that parameter’s change was common for the involved primings.

In summary, this study corroborates that seed priming with PAs, not only the usual ones (Put) but also the uncommon such as DAP, can be an efficient technological approach for plant hardening and improving plant stress tolerance. The efficiency of the priming depends on the changes that the priming agent induces in the part of the Glu metabolism related to the PA synthesis, with AcOrn, Orn, and Cit as crucial players regulating plant performance under optimal and stress conditions (Figure 7). However, the seed priming with Orn induced contrasting responses, mainly 1 mM Orn inhibited plant growth. Two possible scenarios can explain this plant reduction: the activation of a conservative strategy to deal better with the stress or the induction of negative stress (distress) in the plant. In this regard, foliar application with Orn to sugar beet (*Beta vulgaris* var. *saccharifera* L.) alleviated the adverse effect of the water deficit by increasing the biomass production and accumulation of photosynthetic pigments, proteins, and total soluble sugars, as well as the antioxidant defense [22]. Further studies will be needed to understand better the effect of the exogenous Orn application. Finally, our results also pointed to the conjugation of PAs as the most relevant change induced by the seed priming. The conjugation could be by binding phenolic compounds or other macromolecules RNA or proteins as post-translation regulation, most likely to determine the resilience of the plants. However, these responses are defined by the interaction between the priming agent concentration and the plant growth conditions.

## 5. Materials and Methods

### 5.1. Plant Material, Priming, and Growth Conditions

Seeds of *Arabidopsis thaliana* (L.) Heynh. (Col-0 ecotype) were surface sterilized with 70% Ethanol plus 0.01% Triton X-100 following the protocol described in [31]. The sterilized seeds were distributed homogeneously with the help of toothpicks autoclaved on a sheet of autoclaved filter paper moistened with sterile water under laminar flux chamber conditions. After 3–4 min, the filter paper with the seeds was transferred to a square plate (120 × 120 mm, P-Lab, Ref. 212358.2) containing sucrose-free half-strength solid Murashige and Skoog (Phytotechlab M519) medium. For the priming treatments, 1,3-diaminopropane (DAP), Ornithine (Orn), and Putrescine (Put) at two final concentrations (0.1 and 1 mM) were added to the medium. Control treatment consisted of the seeds sown in the square plates with the plain medium. After the sowing, the square plates were led with micro-pore tape and kept in the dark at 4 °C for 4 days. The plates were then positioned vertically in the growth chamber under controlled conditions: temperature of 22 °C, 60% relative humidity, 16/8 h (light/dark), and 120 μmol photons s^−1^ m^−2^ [28] for five days.

### 5.2. Plant Growth under Optimal Conditions and Osmotic and Salt Stresses

Three days after germination, seedlings of similar size were manually transferred, under laminar flux chamber conditions, into 48 and 24-well plates (Jetbiofil, Guangzhou, China), 1 plant per well. Each of the 48 and 24-well plates was previously filled with 1× MS growth medium (pH 5.7; supplemented with 0.6% Phytagel) for the optimal growing condition. The growth medium was supplemented with Mannitol (osmotic stress) and NaCl (salt stress), both at 100 mM for the stress conditions. After the transfer, the well plates were sealed with transparent film, manually perforated to allow the water and gas exchange and avoid water condensation that might have made the image analysis difficult. Finally, the sealed well plates with plants were transferred to the OloPhen platform “Olophen. Available online: http://www.plant-phenotyping.org/db_infrastructure#/tool/57 (Accessed on 10 March 2021)”, consisting of the PlantScreenTM XYZ system where the growth conditions were set to simulate a long day (16/8 h light/dark cycle) with a temperature regime of 20/22 °C, an irradiance of 120 μmol photons s^−1^ m^−2^, and relative humidity of 60%.

### 5.3. Plant Phenotyping Determinations

The phenotyping platform is placed within a controlled conditions growth chamber (cold-white and far-red LED lighting system, temperature, and humidity) (Photon Systems Instruments, Brno, Czech Republic). The system consists of a robotically driven arm lodging an RGB camera with a customized lighting panel and a table where the plates are located. The robotic arm moves above the plates, taking top view pictures [28]. The robotic arm was programmed to acquire RGB images twice per day (10:00 and 16:00) for seven consecutive days, as described in [31]. The RGB camera (top-view; resolution 2560 × 1920) was located 20 cm high on the well-plate. The outcome is the individual picture of the 48 Arabidopsis seedlings per treatment (genotype vs. priming vs. growth condition) as biological replicates for the analyzed phenotypical traits.

#### 5.3.1. Rosette Growth Analysis

The images were stored and analyzed to extract the underlying information using the software described in a previous report [28] “GITHUB, Available online, https://github.com/UPOL-Plant-phenotyping-research-group/In-vitro-plant-growth-analyzer (accessed on 15 March 2021). After the image processing, the leaf rosettes of the seedlings were segmented, and the number of pixels corresponding to each plant was taken as the indicator of the plant area. From this data, different traits were calculated: the slope of *A. thaliana* rosette growth curves (GC-slope) and the area under the curve (AUC), the relative growth ratio (RGR), and the final size of the rosettes at the end of the experiment (FG):

AUC = ∑((s*_i_* − s_(*i*−1)_))/2

where s*_i_* the rosette size at the moment *i*, and s_(*i*−1)_ the rosette size in the immediate previous measurement:

RGR = [(ln(s_2_) − ln(s_1_))/(t_2_ − t_1_)]

where s_1_ and s_2_ are the initial and final size, respectively; and t_1_ and t_2_ are the initial and final time, respectively.

All those traits were used to characterize the behavior of the different genotypes under stress and the influence of the priming, and its combination allowed the calculation of the Plant Biostimulant Characterization Index (PBCI) [31]. The PBCI is the sum of the number obtained by the log_2_ of the ratio between the treatment (compound and concentration) and the controls (umprimed seeds) at each growth condition for each phenotyping trait.

#### 5.3.2. Rosette Color Indices

The software used for the segmentation also provides valuable information about the particular color channels red (R), green (G), and blue (B) extracted from each pixel within the plant mask. the greenness of the *A. thaliana* seedlings and possible change in leaf color index (GLI) due to stressors and/or treatments, which has been correlated with plant biomass, nutrient status, and abiotic stress [52,53,54], was calculated using the equation:

GLI = [(2G − R − B)/(2G + R + B)]

### 5.4. Targeted Metabolomic Analysis

At day 7 of growth, the Arabidopsis rosettes of all variants were harvested, snap-frozen in liquid nitrogen, and stored at −80 °C. The samples were lyophilized, and the obtained DW was used for the targeted metabolomic analysis. The analysis of the free amino acids and total and free polyamines was performed as described by [42]. Briefly, for the analysis of free amino acids, pulverized plant material (3–5 mg) was mixed with 1 mL of 50% EtOH and sonicated for 10 min (Bandelin, Germany). After centrifugation (Prism, Labnet, St. Louis, MO, USA) at 14,500× *g*, the supernatant was transferred into the new vial and kept at 20 °C until analysis. A 200 μL of supernatant was evaporated to dryness and redissolved in the mobile phase. For the quantification, UHPLC-MS/MS analysis was performed on Nexera X2 UHPLC (Shimadzu Handels GmbH, Kyoto, Japan), coupled with MS-8050 (ShimadzuHandels GmbH, Kyoto, Japan). Chromatographic separation was performed on an Acquity UPLC BEH AMIDE (50 *×* 2.1 mm; 1.7-μm particle size) with an appropriate pre-column. All target amino acids were separated using a binary gradient consisting of 15 mM formic acid, pH 3 (component A), and 0.1% formic acid in ACN (component B).

For the polyamines, 200 μL of 2 M NaOH was added to 200 μL of supernatant from the amino acid extraction, followed by 2.5 μL of benzoyl chloride (in MeOH, 50:50, *v:v*), and after vortexing for 5 s, the reaction mixture was stirred for 40 min at 25 °C. About 500 μL of saturated NaCl was added, and benzoylated polyamines were extracted with 2 μL x 500 μL of diethyl ether. The solvent was evaporated under the vacuum at 40 °C, and dry samples were dissolved in 200 μL of the mobile phase and analyzed according to the method described before by [39]. To quantify total polyamines, 200 μL of supernatant was acidified with 50 μL of conc. HCl and shaken for 16 h at room temperature. The 200 μL of 6 M NaOH was added, and derivatization was performed as described above.

### 5.5. Data Analysis

Kruskal–Wallis was used to determine the differences in the AUC of the plants’ growth, with the Infostat software. Multi-ANOVA was performed to determine the differences in the plant metabolites. Data were log-transformed to normalize them before analysis. Duncan’s test was used as a post hoc test for the multiple comparisons between the variants. Multivariate statistical analysis was also carried out. Principal component analysis (PCA) was conducted using singular value decomposition, and PCA biplots were constructed. Heatmaps with dendrograms were produced. Pearson correlations were computed and displayed. All analyses were performed in RStudio (R Software version 4.1.0).

## Data Availability

Not applicable.

## Supplementary Materials

The following supporting information can be downloaded at https://www.mdpi.com/article/10.3390/plants11101287/s1. Table S1: Phenotyping traits in Arabidopsis seedlings from primed seeds with Put, Orn, or DAP at two concentrations (1 or 0.1 mM) under different growth conditions. Table S2: Content of free amino acid in Arabidopsis seedlings from primed seeds with Put, Orn, or DAP at two concentrations (1 or 0.1 mM) under different growth conditions. Table S3: Content of total and free polyamines in Arabidopsis seedlings from primed seeds with Put, Orn, or DAP at two concentrations (1 or 0.1 mM) under different growth conditions.
