# Supplementary material for: Priming with Small Molecule-Based Biostimulants to Improve Abiotic Stress Tolerance in Arabidopsis thaliana
Source: Plants (Basel). 2022 May 11;11(10):1287. doi: 10.3390/plants11101287 (PMC9144751; doi:10.3390/plants11101287)
Supplement: Supplementary file 1 [file plants-11-01287-s001.zip › plants-1723603-supplementary.pdf]

Supplementary Table S1. Phenotyping traits in Arabidopsis seedlings from primed seeds with Put, Orn or DAP at two concentrations (1 or 0.1 mM) under different growth conditions. Area under the growth curve (AUC), early seedling establishment (ESE), the slope of the growth curve (Slope), relative growth ratio (RGR), final growth (FG), and color index (GLI).

		**Optimal**		**Osmotic stress**		**Salt stress**	
**Trait**	**Priming**	**Mean ± s.e.**	**n**	**Mean ± s.e.**	**n**	**Mean ± s.e.**	**n**
**AUC**	**Non**	4800.7 ± 204.81	48	2213.3 ± 150.4	48	2013.4 ± 73.66	48
	**DAP 0.1**	5059.1 ± 200.56	48	2052.7 ± 91.27	48	2147.1 ± 102.54	48
	**DAP 1**	4920.9 ± 268.08	48	2438.4 ± 162.34	48	3062.7 ± 126.33	48
	**Orn 0.1**	4550.9 ± 232.6	48	2322.8 ± 127.7	48	2508.6 ± 104.96	48
	**Orn 1**	3285 ± 164.5	48	1710.1 ± 87.95	48	1708.1 ± 93.9	48
	**Put 0.1**	4595.6 ± 222.78	48	2184.1 ± 115.08	48	2606.4 ± 111.53	48
	**Put 1**	4420.9 ± 210.87	48	2264.4 ± 140.09	48	2291.5 ± 86.41	48
**ESE**	**Non**	276.57 ± 10.9	46	211.41 ± 11.57	44	203.46 ± 8.31	48
	**DAP 0.1**	279.35 ± 12.92	46	211.91 ± 9.08	45	237.57 ± 9.59	46
	**DAP 1**	320.77 ± 14.34	48	231.02 ± 9.81	45	264.15 ± 12.38	47
	**Orn 0.1**	274.63 ± 11.66	48	213.35 ± 9.27	43	259.02 ± 11.66	46
	**Orn 1**	189.65 ± 8.7	43	173.98 ± 7.21	40	193.23 ± 9	43
	**Put 0.1**	282.98 ± 10.2	45	182.3 ± 7.94	46	259.98 ± 11.43	48
	**Put 1**	267.7 ± 13.65	46	217.96 ± 9.39	45	242.28 ± 8.85	46
**Slope (x 10^{-3})**	**Non**	17.13 ± 0.42	47	10.26 ± 0.57	47	7.77 ± 0.43	46
	**DAP 0.1**	17.26 ± 0.37	48	9.29 ± 0.48	47	7.65 ± 0.37	48
	**DAP 1**	15.42 ± 0.39	47	11.05 ± 0.49	46	10.82 ± 0.44	48
	**Orn 0.1**	15.31 ± 0.62	48	11.36 ± 0.46	47	7.94 ± 0.36	48
	**Orn 1**	16.14 ± 0.65	48	10.32 ± 0.5	47	7.81 ± 0.39	46
	**Put 0.1**	16.01 ± 0.51	47	12.46 ± 0.47	47	8.45 ± 0.45	48
	**Put 1**	16.64 ± 0.57	47	10.3 ± 0.5	47	7.9 ± 0.35	47
**RGR**	**Non**	0.413 ± 0.008	46	0.247 ± 0.014	43	0.214 ± 0.011	46
	**DAP 0.1**	0.42 ± 0.009	46	0.228 ± 0.011	42	0.2 ± 0.01	45
	**DAP 1**	0.374 ± 0.011	47	0.273 ± 0.013	42	0.277 ± 0.011	47
	**Orn 0.1**	0.373 ± 0.016	48	0.284 ± 0.011	43	0.214 ± 0.009	46
	**Orn 1**	0.409 ± 0.013	43	0.256 ± 0.013	39	0.193 ± 0.011	43
	**Put 0.1**	0.383 ± 0.015	45	0.306 ± 0.01	46	0.228 ± 0.011	48
	**Put 1**	0.398 ± 0.013	45	0.254 ± 0.012	45	0.215 ± 0.009	44
**FG**	**Non**	3297.3 ± 149.9	48	963.2 ± 67.9	48	706.9 ± 32.5	48
	**DAP 0.1**	3314.9 ± 150.9	48	822.3 ± 44.8	48	815.7 ± 53.4	48
	**DAP 1**	3013.9 ± 161.4	48	1122.7 ± 69.4	48	1290 ± 80.2	48
	**Orn 0.1**	2922 ± 181.5	48	1075.6 ± 62.6	48	863.8 ± 44.2	48
	**Orn 1**	2215.1 ± 130.3	48	793.8 ± 42.9	48	636.1 ± 39.2	48
	**Put 0.1**	3002.9 ± 165.9	48	1103.4 ± 56	48	1051.5 ± 66.4	48
	**Put 1**	2849 ± 156.4	48	996 ± 66.1	48	848.1 ± 49.2	48
**GLI (x 10^{-2})**	**Non**	12.5 ± 0.18	48	9 ± 0.38	48	6.4 ± 0.33	48
	**DAP 0.1**	12.8 ± 0.18	48	7.8 ± 0.23	46	7.1 ± 0.26	48
	**DAP 1**	12.6 ± 0.31	48	9 ± 0.34	47	8.6 ± 0.32	47
	**Orn 0.1**	11.7 ± 0.41	48	10.1 ± 0.26	47	7.9 ± 0.28	48
	**Orn 1**	11.5 ± 0.32	47	8.4 ± 0.3	48	6.7 ± 0.31	48
	**Put 0.1**	12 ± 0.28	48	9.7 ± 0.26	48	8.2 ± 0.28	48
	**Put 1**	11.7 ± 0.33	48	9 ± 0.28	47	7.9 ± 0.34	48

Supplementary Table S2. Content of free amino acid (pmol mg^{-1} DW, except AcPut in fmol mg^{-1} DW) in Arabidopsis seedlings from primed seeds with Put, Orn or DAP at two concentrations 1 or 0.1 mM under different growth conditions. Mean ± s.e. N= 4.

	Priming	Tyra	Leu	Ile	Phe	AcPut	GABA	Trp	BABA	Met	Val	Pro	B-Ala	Tyr	Ala	Thr	AcOrn	AAA	Gly	Asp	Glu	Ser	Asn	Gln	Cit	Arg	His	Orn	Cis
Optimal conditions	Non	0.02 ± 0.01	0.52 ± 0.10	0.47 ± 0.18	0.45 ± 0.07	0.17 ± 0.02	38.51 ± 7.14	0.35 ± 0.11	1.73 ± 0.86	0.34 ± 0.11	29.08 ± 8.02	335.47 ± 46.98	23.73 ± 4.75	3.41 ± 0.56	256.19 ± 25.77	128.01 ± 22.11	1.94 ± 1.06	1.78 ± 0.48	210.23 ± 21.27	1107.06 ± 302.13	3.5 ± 0.96	209.3 ± 36.7	33.91 ± 11.7	541.61 ± 191.42	118.25 ± 16.88	64.89 ± 6.96	4.62 ± 1.56	16.01 ± 1.62	0.48 ± 0.28
	DAP 0.1	0.05 ± 0.02	1.77 ± 0.39	1.00 ± 0.30	2.16 ± 1.70	0.89 ± 0.34	52.75 ± 18.08	2.48 ± 0.94	9.72 ± 5.02	3.89 ± 1.74	29.98 ± 14.47	1796.39 ± 407.75	31.84 ± 8.5	8.45 ± 4.96	244.08 ± 81.63	61.6 ± 8.92	42.03 ± 9.21	1.61 ± 1.06	128.63 ± 29.47	866.18 ± 794.44	23.04 ± 15.86	791.47 ± 298.34	126.24 ± 55.98	2853.51 ± 816.11	79.13 ± 20.51	226.68 ± 75.54	7.28 ± 1.91	13.39 ± 2.38	0.63 ± 0.22
	DAP 1	0.08 ± 0.02	2.1 ± 0.59	1.45 ± 0.21	1.54 ± 0.32	0.85 ± 0.29	103.08 ± 20.55	1.5 ± 0.19	27.5 ± 7.59	6.87 ± 1.53	46.95 ± 9.12	665.64 ± 114.05	28.19 ± 3.36	6.23 ± 1.06	367.69 ± 51.3	196.58 ± 19.06	28.41 ± 5.93	1.54 ± 0.56	231.61 ± 43.92	1141.6 ± 402.79	7.03 ± 1.39	278.31 ± 44.88	51.83 ± 10.85	1241.23 ± 307.31	149.62 ± 26.86	124.34 ± 21.52	13.07 ± 2.7	16.05 ± 2.21	0.06 ± 0.02
	Orn 0.1	0.05 ± 0.01	1.63 ± 0.43	1.08 ± 0.40	1.34 ± 0.27	0.77 ± 0.22	49.25 ± 11.85	1.64 ± 0.26	6.87 ± 2.29	9.75 ± 2.05	52.49 ± 10.24	737.51 ± 134.09	29.42 ± 5.45	6.28 ± 1.27	422.86 ± 86.22	175.75 ± 24.04	21.9 ± 3.51	1.28 ± 0.11	274.04 ± 63.01	1606.29 ± 214.43	1.77 ± 0.2	159.69 ± 42.59	22.82 ± 6.71	1045.09 ± 246.13	101.71 ± 15.04	102.27 ± 17.06	9.83 ± 2.03	10.51 ± 1.76	0.04 ± 0.01
	Orn 1	0.03 ± 0.00	1.51 ± 0.18	1.08 ± 0.09	1.29 ± 0.13	2.23 ± 0.64	58.56 ± 12.27	0.99 ± 0.12	4.58 ± 0.19	2.51 ± 0.18	30.47 ± 1.33	694.96 ± 173.07	28.6 ± 3.41	5.46 ± 0.95	246.97 ± 21.57	89.37 ± 6.56	34.61 ± 7.59	0.71 ± 0.08	157.52 ± 24.39	384.38 ± 69.62	15.35 ± 1.96	586.32 ± 53.59	91.1 ± 12.52	1707.1 ± 370.68	97 ± 9.15	147.5 ± 22.73	4.19 ± 1.58	8.92 ± 2.07	0.05 ± 0.02
	Put 0.1	0.06 ± 0.01	1.02 ± 0.40	0.85 ± 0.32	1.12 ± 0.23	0.84 ± 0.31	23.47 ± 7.96	1.08 ± 0.55	7.78 ± 2.13	3.21 ± 0.83	40.35 ± 14.69	600.83 ± 72.76	23.03 ± 4.58	5.1 ± 0.94	286.33 ± 65.94	138.73 ± 30.93	23.51 ± 11.35	0.93 ± 0.3	237.28 ± 32.59	332.07 ± 109.69	2.67 ± 1.07	169.65 ± 49.05	23.53 ± 8.47	1166.7 ± 281.17	97.79 ± 25.65	104.95 ± 24.29	6 ± 1.19	10.71 ± 3.23	0.02 ± 0.00
	Put 1	0.02 ± 0.00	1.36 ± 0.37	1.07 ± 0.30	0.74 ± 0.14	0.17 ± 0.03	12.68 ± 2.31	0.39 ± 0.39	5.74 ± 1.78	1.6 ± 0.64	45.24 ± 11.67	385.68 ± 65.84	6.19 ± 1.26	3.74 ± 0.95	101.75 ± 25.55	152.83 ± 46.91	7.32 ± 1.72	1.07 ± 0.39	87.78 ± 15	513.81 ± 159.4	42.3 ± 8.55	518.03 ± 70.85	153.48 ± 38.85	846.69 ± 171.8	40.7 ± 9.64	96.4 ± 19.5	7.17 ± 1.15	7.44 ± 2.09	0.02 ± 0.01
Osmotic stress	Non	0.02 ± 0.01	1.68 ± 0.59	1.46 ± 0.28	1.9 ± 0.27	0.57 ± 0.25	28.58 ± 7.58	1.56 ± 0.35	4.24 ± 1.2	4.58 ± 1.7	41.87 ± 11.34	492.89 ± 109.53	9.32 ± 3.41	4.59 ± 1.01	136.43 ± 24.76	150.63 ± 26.58	9.06 ± 2.4	0.88 ± 0.22	147.28 ± 18.46	344.46 ± 78.17	147.78 ± 30.2	823.69 ± 291.65	279.96 ± 112.1	937.65 ± 169.26	47.29 ± 10.77	106.12 ± 29.69	4.8 ± 1.06	9.49 ± 1.8	0.28 ± 0.06
	DAP 0.1	0.03 ± 0.01	1.41 ± 0.40	1.17 ± 0.33	0.77 ± 0.19	0.27 ± 0.09	32.83 ± 11.41	1.63 ± 0.96	6.75 ± 1.38	1.1 ± 0.61	36.44 ± 5.55	415.46 ± 108.6	10.55 ± 1.95	4.6 ± 0.54	111.74 ± 22.44	114.07 ± 30.71	5.25 ± 2.17	0.99 ± 0.22	86.73 ± 17.91	50.47 ± 8.63	47.66 ± 8.06	453.33 ± 48.95	138.58 ± 20.19	1294.55 ± 214.18	21.09 ± 2.21	61.58 ± 13.76	5.72 ± 0.79	3.87 ± 0.71	0.32 ± 0.08
	DAP 1	0.04 ± 0.01	1.38 ± 0.32	0.97 ± 0.15	1.12 ± 0.26	0.68 ± 0.10	52.58 ± 9.39	1.59 ± 0.25	3.26 ± 0.44	1.92 ± 0.32	20.25 ± 3.02	683.27 ± 127.72	11.11 ± 1.91	4.71 ± 0.7	127.95 ± 29.51	107.25 ± 25.46	2.69 ± 0.84	0.41 ± 0.08	69.47 ± 19.13	245.47 ± 66.03	38.11 ± 13.91	510.07 ± 93.69	113.45 ± 34.73	1506.48 ± 296.14	19.14 ± 4.15	64.51 ± 9.5	7.47 ± 1.22	3.19 ± 0.73	0.04 ± 0.01
	Orn 0.1	0.06 ± 0.00	1.74 ± 0.13	1.42 ± 0.10	1.53 ± 0.14	0.74 ± 0.05	39.86 ± 13.77	1.83 ± 0.26	5.32 ± 1.03	1.23 ± 0.16	24.26 ± 1.43	949.14 ± 147.16	12.03 ± 1.12	6.21 ± 0.2	153.91 ± 25.9	157.51 ± 25.71	5.21 ± 1.02	0.55 ± 0.1	97.02 ± 19.94	335.65 ± 63.72	34.85 ± 10.55	549.23 ± 129.47	72.01 ± 19.83	2142.54 ± 227.59	27.45 ± 3.3	128.6 ± 27.21	7.09 ± 1.59	6.1 ± 1.13	0.08 ± 0.02
	Orn 1	0.05 ± 0.02	0.66 ± 0.28	0.46 ± 0.17	0.79 ± 0.27	0.19 ± 0.10	7.13 ± 2.41	1.07 ± 0.43	7.76 ± 3.18	0.87 ± 0.36	16.82 ± 3.49	343.42 ± 117.81	3.34 ± 1.14	2.17 ± 0.98	68.3 ± ± 3.56	59.87 ± 20.41	1.38 ± 0.34	0.66 ± 0.27	51.8 ± 16.51	37.08 ± 9.3	[illegible]00.73 ± 35.2[illegible]	1777.41 ± 128.78	184.36 ± 44.51	1411.57 ± 364.67	28.41 ± 10.4	112.63 ± 43.65	2.16 ± 0.37	3.69 ± 1.35	0.03 ± 0.02
	Put 0.1	0.08 ± 0.02	4.92 ± 1.18	2.86 ± 0.19	2.71 ± 0.59	0.07 ± 0.04	115.06 ± 23.69	0.04 ± 0.00	11.3 ± 1.43	4.37 ± 1.7	60.8 ± 4.6	3138.18 ± 497.01	39.69 ± 5.89	15.71 ± 3.08	501.66 ± 50.08	281.59 ± 36.3	15.18 ± 2.15	1.08 ± 0.25	97.91 ± 16.19	500.65 ± 33	22.3 ± 3.89	1864.69 ± 147.17	24.21 ± 4.61	1150.52 ± 247.25	113.2 ± 6.52	140.25 ± 18.84	3.91 ± 1.08	37.75 ± 11.1	0.01 ± 0.00
	Put 1	0.06 ± 0.02	1.07 ± 0.26	0.85 ± 0.20	1.01 ± 0.30	0.57 ± 0.15	27.9 ± 5.48	0.002 ± 0.00	8.72 ± 1.51	0.96 ± 0.31	24.77 ± 4.27	581.74 ± 154.12	7.56 ± 1.65	3.76 ± 0.78	133.91 ± 31.51	135.43 ± 34.52	3.41 ± 0.71	0.47 ± 0.13	121.04 ± 29.52	246.4 ± 68.11	20.6 ± 1.94	138.75 ± 29.32	34.82 ± 7.9	652.26 ± 161.81	18.97 ± 4.43	51.06 ± 13.26	4.5 ± 1.51	4.14 ± 1.27	0.01 ± 0.01
Salt stress	Non	0.04 ± 0.02	2.33 ± 1.16	1.28 ± 0.64	1.53 ± 0.77	1.77 ± 0.89	125.97 ± 62.99	4.3 ± 2.15	3.62 ± 1.81	1.93 ± 0.97	48.25 ± 24.13	1529.89 ± 764.95	23.94 ± 11.97	5.18 ± 2.59	307.14 ± 153.57	158.88 ± 79.44	7.16 ± 3.58	1.57 ± 0.79	164.69 ± 82.34	345.62 ± 172.81	45.76 ± 22.88	1811.59 ± 905.8	79.35 ± 39.68	920.07 ± 460.03	158.12 ± 79.06	123.64 ± 61.82	8.55 ± 4.27	31.18 ± 15.59	0.24 ± 0.12
	DAP 0.1	0.06 ± 0.03	1.98 ± 0.54	1.01 ± 0.32	1.39 ± 0.28	0.14 ± 0.03	44.48 ± 14.25	0.71 ± 0.29	4.26 ± 0.6	1.11 ± 0.35	24.35 ± 2.95	1683.7 ± 331.45	21.38 ± 4.32	4.12 ± 1.73	205.98 ± 42.62	231.2 ± 46.18	137.27 ± 26.28	1.03 ± 0.13	48.49 ± 16.61	128.46 ± 27.65	30.62 ± 7.92	1923.89 ± 586.38	31.19 ± 9.26	759.98 ± 199.61	98.15 ± 26.35	96.65 ± 21.62	3.77 ± 0.76	32.58 ± 6.83	0.14 ± 0.06
	DAP 1	0.03 ± 0.02	1.74 ± 0.43	0.69 ± 0.17	0.99 ± 0.16	0.76 ± 0.45	51.58 ± 14.98	1.05 ± 0.37	4.44 ± 0.77	3.19 ± 1.29	22.56 ± 4.4	1471.65 ± 227.68	22.93 ± 5.96	6.71 ± 1.17	232.15 ± 93.67	209.46 ± 54.6	115.13 ± 15.74	0.88 ± 0.27	38.29 ± 9	159.02 ± 57.27	43.82 ± 12.05	3223.58 ± 837.29	33.72 ± 5.37	394.03 ± 56.35	50.86 ± 10.37	51.92 ± 10.37	5.21 ± 1.05	7.68 ± 2.48	0.03 ± 0.01
	Orn 0.1	0.02 ± 0.00	1.71 ± 0.25	1.01 ± 0.15	1.43 ± 0.15	0.11 ± 0.06	48.85 ± 14.97	0.92 ± 0.16	5.51 ± 1.86	1.77 ± 0.69	68.15 ± 19.38	1834.87 ± 179.32	30.86 ± 4.43	7.46 ± 1.77	530.22 ± 162.79	273.07 ± 65.51	200.31 ± 17.32	2.4 ± 1.01	120.92 ± 41.89	1060.16 ± 560.62	19.62 ± 2.89	1215.33 ± 133.98	46.98 ± 6.7	1215.46 ± 207.37	200.74 ± 21	132.05 ± 16.61	2.29 ± 0.46	23.86 ± 5.79	0.03 ± 0.01
	Orn 1	0.02 ± 0.01	1.58 ± 0.58	1.18 ± 0.49	1.53 ± 0.61	0.04 ± 0.01	10.26 ± 3.27	0.84 ± 0.23	8.12 ± 1.79	2.05 ± 1.07	42.48 ± 12.35	419.19 ± 154.31	21.75 ± 5.21	3.23 ± 1.38	283.99 ± 63.57	258.83 ± 33.5	53.81 ± 9.44	2.76 ± 0.87	168.19 ± 8.33	442.55 ± 128.85	[illegible]13.49 ± 41.8[illegible]	4205.75 ± 1983.27	128.57 ± 54.69	642.81 ± 216.28	129.56 ± 33.02	107.43 ± 38.14	4.43 ± 1.47	35.02 ± 8.39	0.01 ± 0.00
	Put 0.1	0.11 ± 0.03	1.90 ± 0.61	1.70 ± 0.37	1.62 ± 0.43	0.99 ± 0.16	42.49 ± 12.66	0.16 ± 0.15	9.73 ± 3.22	2.06 ± 0.69	36.92 ± 6.57	543.07 ± 140.56	11.12 ± 3.49	4.45 ± 1.01	138.85 ± 27.53	140.81 ± 32.53	3.27 ± 1.3	0.53 ± 0.13	104.33 ± 24.9	436.47 ± 118.38	15.94 ± 6.59	195.95 ± 76.91	113.92 ± 32.22	782.18 ± 186.59	19.89 ± 4.91	80.66 ± 34.69	6.88 ± 1.23	2.6 ± 0.63	0.04 ± 0.02
	Put 1	0.03 ± 0.01	1.60 ± 0.50	0.86 ± 0.33	1.28 ± 0.24	0.46 ± 0.24	30.39 ± 8.59	0.02 ± 0.01	6.05 ± 2.6	0.63 ± 0.22	32.8 ± 4.44	2128.18 ± 160.54	26.77 ± 1.76	5.97 ± 1.98	382.28 ± 143.4	416.91 ± 68.38	132.17 ± 35.25	1.37 ± 0.47	288.09 ± 64.65	1571.26 ± 662.57	61.09 ± 49.69	1090.05 ± 221.02	26.74 ± 3.91	513.69 ± 148.09	165.41 ± 27.74	89.43 ± 28.71	1.61 ± 0.74	104.72 ± 16.66	0.02 ± 0.00

Supplementary Table S3. Content of total and free polyamines (pmol mg^{-1} DW) in Arabidopsis seedlings from primed seeds with Put, Orn or DAP at two concentrations 1 or 0.1 mM under different growth conditions. Mean ± s.e. N= 4

		Total polyamines (conjugated +free)									
		**Hist**	**Put**	**DAP**	**Cad**	**NorSpd**	**Spd**	**HomoSpd**	**ThSpm**	**Spm**	**Agm**
**Optimal conditions**	**Non**	**<LOD**	1483.92 ± 622.92	47.46 ± 21.07	2.09 ± 0.77	82.66 ± 15.3	3947.72 ± 890.64	66.64 ± 31.02	11.56 ± 3.96	576.62 ± 43.46	784.72 ± 300.69
	**DAP 0.1**	**<LOD**	2946.58 ± 646.06	130.97 ± 38.35	7.77 ± 8.07	122.64 ± 17.39	5391 ± 883.97	149.99 ± 29.3	18.59 ± 7.05	722.4 ± 179.1	1081.27 ± 219.61
	**DAP 1**	**<LOD**	4381.49 ± 817.72	632.54 ± 155.01	17.18 ± 9.04	139.5 ± 30	7369.9 ± 2860.27	59.77 ± 19.7	47.38 ± 16.06	1510.39 ± 667.16	2497.56 ± 295.92
	**Orn 0.1**	**<LOD**	3368.87 ± 537.14	85.87 ± 51.2	2.53 ± 0.31	238.21 ± 84.36	7015.61 ± 1094.65	61.01 ± 8.88	25.98 ± 11.93	1063.56 ± 251.19	2458.41 ± 299.05
	**Orn 1**	**<LOD**	411.66 ± 238.5	49.69 ± 16.4	2.6 ± 1.09	169.54 ± 84.32	1665.05 ± 445.13	48.61 ± 27.34	4.83 ± 2.27	286.34 ± 32.87	1390.05 ± 351
	**Put 0.1**	**<LOD**	2031.61 ± 457.04	163.81 ± 72.74	7.89 ± 3.42	2919.27 ± 1815.43	5045.13 ± 1901.83	71.6 ± 2.87	11.2 ± 4.4	540.04 ± 218.45	1969.78 ± 491.05
	**Put 1**	**<LOD**	3569.84 ± 1289.46	81.75 ± 32.08	8.13 ± 4.61	132.97 ± 22.77	7516.11 ± 1973.98	61.73 ± 19.55	44.96 ± 9.04	2745.76 ± 2797.11	4692.94 ± 1106.08
**Osmotic stress**	**Non**	**<LOD**	530.01 ± 47.62	42.74 ± 3.98	6.1 ± 3.21	116.77 ± 29.36	3246.52 ± 716.23	56.8 ± 13.74	4.53 ± 1.52	1370.39 ± 426.49	1793.78 ± 843.04
	**DAP 0.1**	**<LOD**	816.28 ± 179.21	140.18 ± 40.22	4.3 ± 1.57	1601.52 ± 365.81	3932.4 ± 1483.99	55.68 ± 17.65	23.36 ± 37.43	1526.91 ± 621.67	2289.2 ± 253.86
	**DAP 1**	**<LOD**	1407.17 ± 348.21	398.14 ± 124.17	15.35 ± 8.18	208.31 ± 105.31	6495.11 ± 794.54	88.36 ± 26.31	11.55 ± 8.92	2371.02 ± 366.85	5171.37 ± 1104.79
	**Orn 0.1**	**<LOD**	793.1 ± 320.42	124.28 ± 57.88	3.58 ± 0.57	122.14 ± 43.03	4727.82 ± 1357.66	77.05 ± 31.11	1.97 ± 0.6	2359.03 ± 718.07	5614.52 ± 1000.09
	**Orn 1**	**<LOD**	211.29 ± 84.81	61.8 ± 18.04	2.9 ± 1.25	722.04 ± 456.34	1544.01 ± 157.39	50.01 ± 12.15	1.86 ± 0.89	713.36 ± 183.38	2066.23 ± 307.06
	**Put 0.1**	**<LOD**	437.53 ± 64.96	89.22 ± 61.95	15.8 ± 5.74	302.5 ± 99.33	2653.87 ± 381.59	68.6 ± 19.25	1.09 ± 0.91	805.63 ± 155.7	2387.28 ± 166.37
	**Put 1**	**<LOD**	972.51 ± 214.69	123.71 ± 67.38	16.45 ± 10.79	3052.22 ± 1383.02	6410.4 ± 1551.01	114.66 ± 63.96	10.28 ± 6.85	4477.64 ± 1500.14	6906.04 ± 1041.27
**Salt stress**	**Non**	**<LOD**	1951.95 ± 644.61	46.04 ± 13.49	2.63 ± 0.71	131.12 ± 23.2	5517.65 ± 1019.01	72.18 ± 13.45	13.4 ± 12.89	111.32 ± 19.54	1875.17 ± 636.91
	**DAP 0.1**	**<LOD**	3067.97 ± 481.52	106.33 ± 29.68	3.81 ± 1.95	168.65 ± 91.49	7591.67 ± 693.42	86.75 ± 21.81	86.49 ± 27.82	2841.42 ± 893.43	1508.86 ± 320.72
	**DAP 1**	**<LOD**	5189.04 ± 698.35	686 ± 160.34	22.37 ± 9.09	91.36 ± 55.48	11413.65 ± 378.39	271.25 ± 16.66	174.09 ± 56.13	4481.67 ± 2698.49	3944.83 ± 1248.91
	**Orn 0.1**	**<LOD**	6448.31 ± 559.55	106.53 ± 29.87	4.38 ± 1.4	103.92 ± 49.45	12705.21 ± 773.56	387.06 ± 242.56	240.04 ± 74.02	5750.77 ± 1613.32	3703.63 ± 1010.48
	**Orn 1**	**<LOD**	2571.39 ± 816.32	61.46 ± 3.52	2.58 ± 0.14	198.86 ± 66.13	9219.48 ± 845.81	81.49 ± 20.53	62.94 ± 20.86	1967.53 ± 632.98	4055.97 ± 1829.44
	**Put 0.1**	**<LOD**	5922.81 ± 918	61.95 ± 32.05	4.6 ± 3	1320.86 ± 130.35	12120.06 ± 2529.84	405.15 ± 210.17	210.03 ± 53.21	4871.3 ± 2506.62	4960.2 ± 1887.06
	**Put 1**	**<LOD**	4474.26 ± 1000.92	82.4 ± 16.16	23.2 ± 13.83	245.65 ± 149.79	11657.04 ± 2615.12	120.37 ± 7.87	212.23 ± 35.09	6943.28 ± 994.38	7291.02 ± 2791.51
		**Free polyamines**									
		**Hist**	**Put**	**DAP**	**Cad**	**NorSpd**	**Spd**	**HomoSpd**	**ThSpm**	**Spm**	**Agm**
**Optimal conditions**	**Non**	39.91 ± 4.76	4362.93 ± 5650.59	47.05 ± 23.47	18.46 ± 4.49	47.24 ± 33.84	819.34 ± 199.55	18.33 ± 8.43	2.47 ± 0.09	23.25 ± 5.37	1004.05 ± 384.27
	**DAP 0.1**	29.22 ± 9.55	1909.48 ± 399	54.39 ± 15.52	15.85 ± 1.1	11.67 ± 5.18	583.94 ± 272.47	20.4 ± 9.48	2.37 ± 0.23	15.96 ± 8.73	625.59 ± 246.49
	**DAP 1**	20.61 ± 5.68	3339.18 ± 345.48	349.56 ± 172.28	40.59 ± 10.56	13.58 ± 2.38	912.8 ± 484.78	8.64 ± 4.99	2.49 ± 0.22	20.57 ± 7.07	953.52 ± 236.03
	**Orn 0.1**	16.56 ± 3.57	2140.54 ± 438.05	23.77 ± 10.47	12.81 ± 3.46	28.28 ± 10.05	655.58 ± 189.46	9.01 ± 2.18	2.14 ± 0.14	14.78 ± 4.79	700.73 ± 221.24
	**Orn 1**	14.88 ± 8.75	471.21 ± 194.48	27.45 ± 20.12	5.57 ± 1.76	21.05 ± 18.74	264.65 ± 120.17	13.44 ± 8.86	2.07 ± 0.04	13.39 ± 8.5	443.43 ± 74.74
	**Put 0.1**	6.81 ± 1.06	1195.08 ± 478.17	91.13 ± 66.7	3.52 ± 1.08	141.85 ± 30.22	628.92 ± 308.97	11.81 ± 3.85	1.13 ± 0.07	13.81 ± 6.11	393.21 ± 194.37
	**Put 1**	54.89 ± 53.49	788.73 ± 92.72	34.42 ± 11.77	31.94 ± 27.96	36.66 ± 30.79	682.57 ± 76.3	9.41 ± 3.41	35.93 ± 29.48	41.3 ± 31.82	1262.51 ± 585.96
**Osmotic stress**	**Non**	34.62 ± 3.43	635.69 ± 277.78	47.4 ± 28.56	17 ± 4.35	33.6 ± 28.2	606.42 ± 44.59	5.68 ± 1.14	2.37 ± 0.25	64.87 ± 17.67	2166.53 ± 447.94
	**DAP 0.1**	44.46 ± 28.95	668.05 ± 172.69	96.77 ± 33.46	18.68 ± 1.47	201.68 ± 83.62	646.78 ± 151.15	4.72 ± 0.75	2.17 ± 0.14	45.36 ± 12.28	1841.61 ± 314.4
	**DAP 1**	41.9 ± 8.22	1345.3 ± 150.18	246.96 ± 71.9	51.23 ± 16.14	41.23 ± 25.2	830.08 ± 528.08	6.68 ± 2.5	2.6 ± 0.54	38.91 ± 16.71	1903.13 ± 496.51
	**Orn 0.1**	13.81 ± 1.73	691.2 ± 282.06	79.3 ± 46.91	10.5 ± 2.27	53.7 ± 51.99	1014.01 ± 464.91	5.1 ± 0.6	2.07 ± 0.05	75.3 ± 61.65	2307.77 ± 482.05
	**Orn 1**	13.7 ± 2.92	338.08 ± 178.68	55.89 ± 12.82	7.65 ± 0.87	101.58 ± 42.42	369.48 ± 143.56	9.97 ± 7.59	2.05 ± 0.05	42.44 ± 24.63	979.95 ± 434.77
	**Put 0.1**	8.32 ± 1.01	201.8 ± 21.97	36.29 ± 27.92	3.25 ± 0.33	16 ± 4.05	339.28 ± 111.92	3.19 ± 0.67	1.04 ± 0.02	31.56 ± 5.93	950.77 ± 333.68
	**Put 1**	18.27 ± 21.03	586.93 ± 241.43	79.51 ± 10.7	13.88 ± 10.99	331.54 ± 236.44	1643.19 ± 499.32	15.78 ± 9.74	2.31 ± 0.28	239.76 ± 138.13	1629.39 ± 717.27
**Salt stress**	**Non**	22.75 ± 4.24	2454.04 ± 478.51	57.2 ± 8.79	22.23 ± 4.29	35.46 ± 38.81	3623.59 ± 606.56	31.92 ± 8.5	5.79 ± 2	78.07 ± 19.25	2214.09 ± 751.84
	**DAP 0.1**	42.34 ± 41.71	3746.55 ± 968.3	51.84 ± 24.52	25 ± 9.01	17.01 ± 3.8	3894.49 ± 845.23	26.58 ± 8.54	4.46 ± 0.29	79.95 ± 13.38	1981.24 ± 544.36
	**DAP 1**	19.82 ± 4.51	4193.5 ± 712.07	403.98 ± 137.08	59.79 ± 13.01	25.07 ± 8.55	4155.99 ± 1786.67	59.56 ± 17.52	4.52 ± 0.99	99.17 ± 16.99	2050.85 ± 392.6
	**Orn 0.1**	28.83 ± 18.62	5304.56 ± 664.69	50.84 ± 13.78	31.24 ± 8.32	32.21 ± 14.97	7049.03 ± 1883.54	26.48 ± 8.24	6.78 ± 1.28	149.47 ± 37.22	2120.45 ± 333.79
	**Orn 1**	15.03 ± 6.02	2545.66 ± 775.17	35.86 ± 8.12	8.87 ± 0.97	30.09 ± 18.27	1928.84 ± 346.31	18.86 ± 9.68	3.37 ± 0.91	47.45 ± 25.41	994.74 ± 385.98
	**Put 0.1**	7.52 ± 2.14	4258.9 ± 1271.57	45.79 ± 17.8	7.32 ± 1.63	10.06 ± 6.78	4160.55 ± 1244.21	70.41 ± 68.98	5.02 ± 2.85	85.4 ± 27.89	1169.07 ± 312.59
	**Put 1**	7.19 ± 7.74	2098.91 ± 355.94	25.55 ± 4.16	8.19 ± 2.54	18.49 ± 7.05	3789.13 ± 894.65	16.52 ± 8.03	4.31 ± 3.26	77.86 ± 25.22	1284.77 ± 314.28
